# Neutrophilia in locally advanced cervical cancer: A novel biomarker for image-guided adaptive brachytherapy?

**DOI:** 10.18632/oncotarget.12440

**Published:** 2016-10-04

**Authors:** Alexandre Escande, Christine Haie-Meder, Pierre Maroun, Sébastien Gouy, Renaud Mazeron, Thomas Leroy, Enrica Bentivegna, Philippe Morice, Eric Deutsch, Cyrus Chargari

**Affiliations:** ^1^ Radiotherapy department, Brachytherapy Unit, Gustave Roussy Cancer Campus, Villejuif, France; ^2^ Faculté de médecine PARIS Sud, université Paris Sud, Université Paris Saclay, France; ^3^ Department of Surgery, Gustave Roussy, Villejuif, France; ^4^ Radiotherapy Department, Oscar Lambret Comprehensive Cancer Center, Lille, France; ^5^ INSERM1030, Gustave Roussy Cancer Campus, Villejuif, France; ^6^ French Military Health Services Academy, Ecole du Val-de-Grâce, Paris, France; ^7^ Institut de Recherche Biomédicale des Armées, Bretigny-sur-Orge, France

**Keywords:** locally advanced cervical cancer, image-guided adaptive brachytherapy, prognostic factor, biomarkers, neutrophilia

## Abstract

**Objective:**

To study the prognostic value of leucocyte disorders in a prospective cohort of cervical cancer patients receiving definitive chemoradiation plus image—guided adaptive brachytherapy (IGABT).

**Results:**

113 patients were identified. All patients received a pelvic irradiation concomitant with chemotherapy, extended to the para-aortic area in 13 patients with IVB disease. Neutrophilia and leukocytosis were significant univariate prognostic factors for poorer local failure-free survival (*p* = 0.000 and *p* = 0.002, respectively), associated with tumor size, high-risk clinical target volume (HR-CTV) and anemia. No effect was shown for distant metastases but leukocytosis and neutrophila were both poor prognostic factors for in-field relapses (*p* = 0.003 and *p* < 0.001). In multivariate analysis, HR-CTV volume (*p* = 0.026) and neutrophils count > 7,500/μl (*p* = 0.018) were independent factors for poorer survival without local failure, with hazard ratio (HR) of 3.1.

**Materials and methods:**

We examined patients treated in our Institution between April 2009 and July 2015 by concurrent chemoradiation (45 Gy in 25 fractions +/− lymph node boosts) followed by a magnetic resonance imaging (MRI)-guided adaptive pulse-dose rate brachytherapy (15 Gy to the intermediate-risk clinical target volume). The prognostic value of pretreatment leucocyte disorders was examined. Leukocytosis and neutrophilia were defined as a leukocyte count or a neutrophils count exceeding 10,000 and 7,500/μl, respectively.

**Conclusions:**

Neutrophilia is a significant prognostic factor for local relapse in locally advanced cervical cancer treated with MRI-based IGABT. This biomarker could help identifying patients with higher risk of local relapse and requiring dose escalation.

## INTRODUCTION

Despite the implementation of the concept of image-guided adaptive brachytherapy (IGABT) and dose escalation, distant failures remain frequent events in locally advanced cervical cancer, occurring in 30–40% of patients and partially alleviating the benefit of improving local control [1–7]. There has been an increasing attempt at better identifying tumor related prognostic factors for locally advanced cervical cancers. Most relevant of these factors include tumor size at diagnosis, FIGO stage, lymph node metastases and volume of high-risk clinical target volume (HR-CTV) [5, 7, 8]. However, it is clear that the ability of these conventional tumor–related risk factors to predict risk of tumor relapse and estimate survival is still insufficient. There is need for more sensitive biomarkers to better determine which patients would get the largest benefit from dose escalation (or at the opposite would be candidates for dose de-escalation), but also to potentially identify patients at very high risk of distant failure requiring intensification of systemic treatments.

Tumor-related leukocytosis (TLR), and more particularly neutrophilia, is a paraneoplastic syndrome reported in various malignant advanced tumors types. It was recently suggested that neutrophilia could be associated with poorer overall survival in primarily treated or recurrent cervical cancer patients [9, 10]. Expression of cancer cell-derived granulocyte-colony stimulating factor (G-CSF) is speculated to be one of the causative mechanisms of neutrophilia, through an aberrant paracrine activity involved in expansion and intra-tumor accumulation of myeloid-derived suppressor cells (MDSCs). MDSCs have been implicated in tumor progression by promoting tumor angiogenesis, metastatic process and immune suppression [11, 12]. Experimentations in murine models have also showed that MDSCs were also associated with higher resistance to radiotherapy [10]. However, the correlation between leucocyte disorders and the risk of local failure has not been examined in locally advanced cervical cancer patients treated according to modern standards.

In the current study, the prognostic significance of systemic leucocyte modifications on survival and on local control was examined in a single center cohort of patients homogeneously treated according to the modern standard based on chemoradiation plus IGABT in the frame of an academic prospective study.

## RESULTS

### Patients and tumors

A total of 113 patients were identified. Median age was 48.3 years (range: 24.7–75.4 years). Histology was squamous cell carcinoma in 95 patients (84.1%). Median largest tumor size was 4.7 cm (range: 1–12 cm). Initial median tumor volume assessed by MRI was 39.7 cm^3^ (range: 10.5–340 cm^3^). Forty-five patients (39.8%) had pelvic nodal uptakes at PET/CT and 13 patients had para-aortic lymph node metastases including five patients with PET positive lymph node metastases and eight patients with histological evidence of para-aortic lymph node metastases. At first week of EBRT, median hemoglobin level was 12.6 g/dL (6.5–15.3 g/dL) and median platelet count was 286 G/L (110–751 G/L). Median leukocytes and neutrophils counts were 8,800/mm^3^ (3,700–22,500/mm^3^) and 5,650/mm^3^ (400–18,700/mm^3^), respectively. Leukocytosis and neutrophilia were found in 28 patients (24.8 %) both. Median lymphocyte count was 1,400/mm^3^ (200–8,200/mm^3^). Neutrophilia at initiation of chemoradiation was associated with a larger tumor at diagnosis (*p* = 0.033), and a larger HR-CTV volume at time of brachytherapy (*p* = 0.012). Characteristics of patients and tumors and blood cell count results are shown in Table 1.

**Table 1:** Patients, tumors and blood cell count characteristics

Characteristics		Median (min-max) or Nb(%)
Number of patients		113 (100)
Age (years)		48.3 (24.7–74.4)
Performance status	0	85 (75.2)
	1	26 (23.0)
	2	2 (1.8)
Histopathological type:	SCC	95 (84.1)
	ADK	18 (15.9)
Tumor volume (cm^3^)		39.7 (10.5–340)
Largest tumor size (cm)		4.7 (1–12)
Maximal size > 5 cm	Yes	61 (46.0)
	No	52 (54.0)
FIGO staging	IB2	33 (29.2)
	IIA	7 (6.2)
	IIB	51 (45.1)
	IIIA	1 (0.9)
	IIIB	3 (2.7)
	IVA	5 (4.4)
	IVB	13 (11.5)
Pelvic nodal metastases	Yes	45 (39.8)
	No	68 (60.2)
Differentiation degree	Well	27 (23.9)
	Moderate	42 (37.2)
	Poor	17 (15)
	ND	27 (23.9)
Blood cell count at first week		
Hemoglobin (g/dl)		12.6 (6.5–15.3)
Platelet (G/L)		286 (110–751)
Leucocytes (/mm^3^)		8,800 (3,700–22,500)
Neutrophils (/mm^3^)		5,650 (400–18,700)
Leucocytosis	Yes	28 (24.8)
	No	85 (75.2)
Neutrophilia	Yes	28 (24.8)
	No	85 (75.2)

ADK: adenocarcinoma; FIGO: International Federation of Gynecology Obstetrics; SCC: squamous cell carcinoma; Nb: number.

### Treatment

All patients received a pelvic irradiation, extended to the para-aortic area in 13 patients with stage IVB disease. All patients received concurrent chemotherapy, consisting of cisplatin in 102 patients (90.3%), carboplatin in six, cisplatin followed by carboplatin because of renal function impairment in five (5.4%). Median dose of sequential boost to macroscopically involved lymph node metastases was 10 Gy (range: 7.2–14 Gy, 1.8–2 Gy per fraction). Median HR-CTV volume was 21.3 cm^3^ (range: 10–79 cm^3^). The median D90 HR-CTV was 38.2 Gy (range: 8.4–50.5 Gy). Ten patients (8.8%) received a parametrial boost using interstitial needles. Median overall treatment time was 47 days (range: 38–62 days). Treatment characteristics are reported in Table 2.

**Table 2:** Treatments characteristics

Characteristics	Median (min-max) or Nb(%)
Number of patients	113 (100)
Radiotherapy	
Dose (Gy)	45
Pelvic irradiation	113 (100)
Para-aortic irradiation	13 (11.5)
Sequential lymph node boosts	45 (39.8)
Median boosts doses (Gy)	10 (7.2–14)
Concurrent chemotherapy Yes	113 (100)
No	0 (0)
Number of chemotherapy courses	6 (3–7)
Chemotherapy type Cisplatine	102 (90.3)
Carboplatine	6 (5.3)
Both	5 (4.4)
Brachytherapy parameters	
MRI guided	113 (100)
HR-CTV volume (cm^3^)	21.3 (9.9–79.0)
HR-CTV D90 (Gy)	38.2 (17.8–50.5)
TRAK	1.73 (0.67–2.43)
Use of interstitial needles	10 (8.8)
Overall treatment time (days)	47 (38–62)

HR-CTV: high-risk clinical target volume; MRI: Magnetic Resonance Imaging; TRAK: Total reference air Kerma.

### Outcome

With median follow-up of 4.1 years (95% CI: 3.47–4.7 years), relapses were reported in 29 patients (25.7%). In details, in-field relapses occurred in 16 patients, including local relapses in 12 patients (10.6%). Out-of field relapses occurred in 22 patients (19.5%), including six patients with para-aortic failure who had not received para-aortic irradiation at time of primary treatment because primary staging showed no para-aortic metastases. At last follow-up, 20 patients (17.7%) had died, all from cancer. At three years, estimated overall survival was 80.0% (95% CI: 75.7–84.3%), estimated relapse-free survival was 73.8% (95% CI: 69.3–78.3%), estimated survival without in-field relapse 85.6% (95% CI: 82–89.2%) and estimated survival without local relapse was 89.0% (95% CI: 85.8– 92.2%)

### Prognostic value of leucocytes disorders

Using univariate analysis, pre-treatment neutrophilia and leukocytosis were significant prognostic factors for local failure-free survival (*p* < 0.001 and *p* = 0.002, respectively), associated with tumor size (*p* = 0.003), HR- CTV volume (*p* = 0.003) and anemia (*p* = 0.036). The effect of leukocytosis or neutrophila was not significant for distant failures but leukocytosis and neutrophila were both poor prognostic factors for in-field relapse (*p* = 0.003 and *p* < 0.001), associated with HR-CTV volume (*p* = 0.008). In univariate analysis significant factors for overall survival were tumor size (*p* = 0.01), HR-CTV volume (*p* = 0.017), neutrophilia (*p* = 0.004) and anemia (*p* = 0.011).

**Figure 1:**
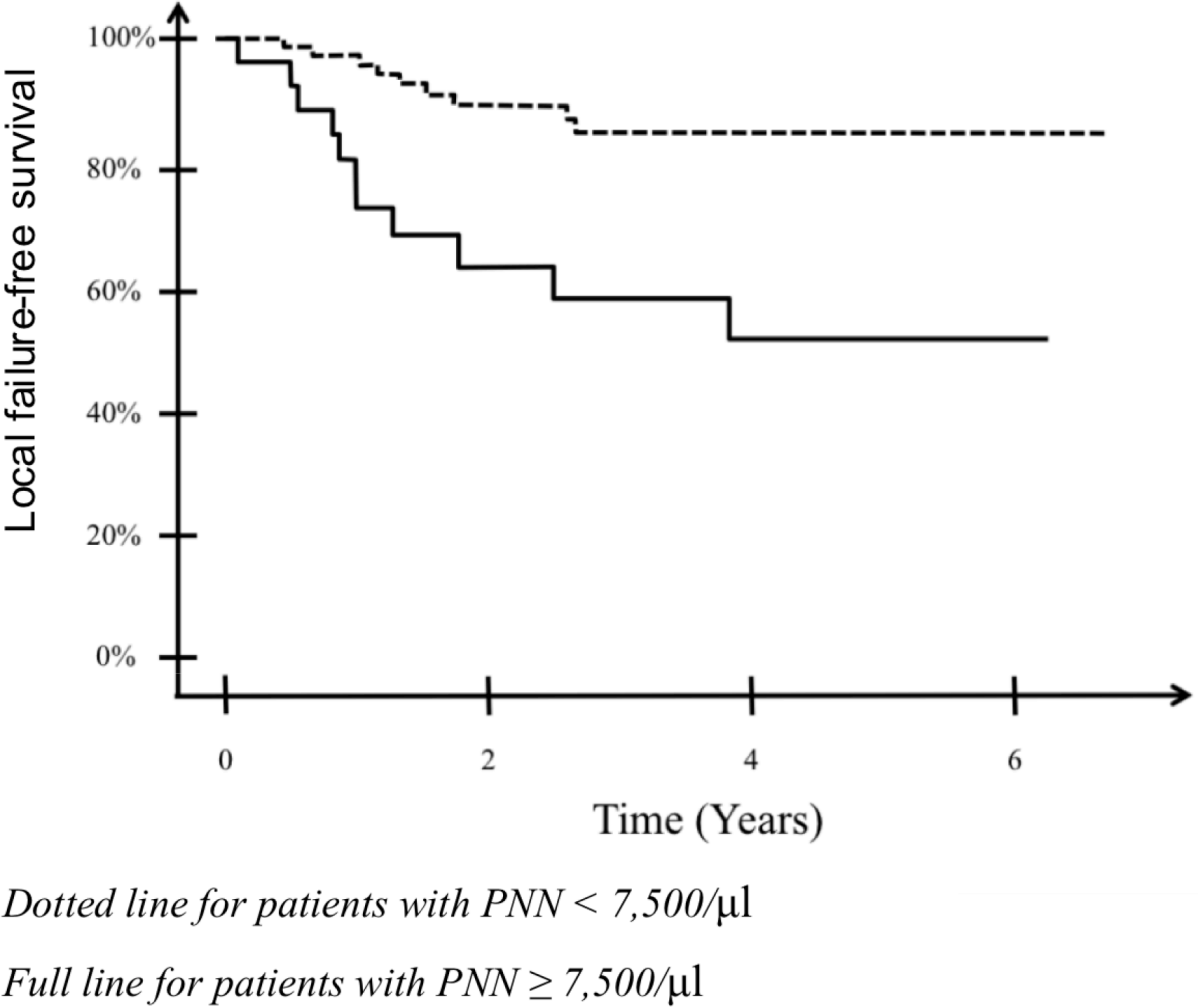
Estimated survival without local failure in patients with or without neutrophilia

Using multivariate analysis, HR-CTV volume (*p* = 0.026) and neutrophils count > 7,500/μl (*p* = 0.018) were independent factors for poorer survival without local failure, with hazard ratio (HR) of 3.1 for both factors. Two prognostic factors were associated with a higher risk of in-field failure: 1/ leukocyte disorders (for neutrophilia: HR = 4.50, *p* = 0.002; for leukocytosis: HR = 2.54, *p* = 0.047) and 2/ a HR-CTV volume > 25cc (HR = 2.94, *p* = 0.034). In multivariate, only HR-CTV volume (*p* = 0.03) and anemia (*p* = 0.026) were significant for overall survival. The significant prognostic value of neutrophilia and leuckocytosis on local-failure free survival remained significant in multivariate analysis after exclusion of patients with stage IVB disease (HR = 8.9 and 6.7, respectively, *p* = 0.002 for both). Results of univariate and multivariate analyses are detailed in Table 3. Local failure-free survival curve according to presence or absence of neutrophilia is shown in Figure 1.

**Table 3:** Results of univariate and multivariate analyses

variable	Overall Survival		In-Field Failure-Free Survival	
	Log-rank	Cox model (HR)	Log-rank	Cox model (HR)
FIGO III-IV	0.959	--	0.552	--
Tumor > 5 cm	0.010	0.512	0.002	0.078
Pelvic LN	0.151	--	0.099	0.288
HR-CTV > 25 cc	0.017	**0.030 (2.86)**	0.002	**0.008 (3.73)**
Neutrophilia^a^	0.004	0.232	0.000	**0.002 (4.50)**
Leucocytosis^a^	0.199	0.962	0.003	**0.047 (2.54)**
Anemia	0.011	**0.026 (3.05)**	0.065	0.233

Variable	Local Failure-Free Survival		Distant Failure-Free Survival	
	Log-rank	Cox model (HR)	Log-rank	Cox model (HR)
FIGO III-IV	0.669	--	0.433	--
Tumor > 5 cm	0.003	0.383	0.013	**0.005 (3.48)**
Pelvic LN	0.914	--	0.047	**0.028 (2.42)**
HR-CTV > 25 cc	0.003	**0.026 (3.1)**	0.075	0.505
Neutrophilia^a^	0.000	**0.018 (3.1)**	0.590	--
Leucocytosis^a^	0.002	0.287	0.564	--
Anemia	0.036	0.177	0.020	0.324

Variables significant with a *p* value < 0.1 in univariate analysis were used for multivariate model (significant factors in multivariate analysis in bold).

FIGO: International Federation of Gynecology Obstetrics; HR: hazard ratio (given only if p < 0.05); HR CTV: high risk clinical target volume; LN: lymph nodes.

a Neutrophilia and leucocytosis were not tested in the same model, as neutrophils are subpopulation of leucocytes.

## DISCUSSION

The progressive implementation of IGABT in locally advanced cervical cancer patients has been associated with a significant increase of local control rates in patients with large tumors at diagnosis [1–7]. Pötter et al. reported their retrospective experience comparing 2D brachytherapy and MRI-guided brachytherapy. The impact of IGABT in tumors less than 5 cm could not be evidenced but for tumors > 5 cm, the use IGABT was associated with an increase in the mean D90 HR-CTV and an improvement of 3-year local control rate from 64% to 82%. The decrease of local relapses was associated with a survival benefit in patients with bulky tumors, from 53% to 64% [3]. In our previously published experience of IGABT, we had reported that the only tumor-related factor associated with local control in multivariate analysis was HR-CTV volume, which reflects a bulky tumor at diagnosis but also poor response to chemoradiation. Analysis of dose-effect curves suggested that doses to the HR-CTV should be increased in higher stage or in patients with a HR-CTV volume > 30 cm^3^ [3]. Although there is sound rationale that dose escalation improves local control in patients with bulky disease, there is no firm demonstration that dosimetric parameters and survival are correlated. It is therefore uncertain whether all patients equally benefit from dose escalation and the possibility that some patients would not benefit from dose escalation cannot be ruled out. Some tumoral factors (FIGO stage, width at diagnosis, treatment time, HR-CTV volume, anemia) have been shown to significantly impact dose volume effect for tumor control, but in the next steps of treatment optimization, other parameters should be taken into account in the treatment planning process, such as tumor biological heterogeneity or tumor response after chemoradiation [5, 7, 8].

Biological parameters could be added to current models for prediction of outcome and tailoring therapeutic strategies based on a personalized assessment of specific risk. Mayr et al. have monitored treatment response by functional MRI and their results suggest that tumor heterogeneity could be characterized by dynamic contrast-enhanced to predict long-term treatment outcome, including the probability of primary tumor control [13]. Anemia is another factor that is frequently reported in the literature, with retrospective analyses suggesting that hemoglobin level during radiotherapy is a strong prognostic factor for local control and survival and a biomarker that could potentially allow identifying poor prognostic subgroups with persisting hypoxia during radiotherapy [14]. The prognostic value of anemia was corroborated in the present study.

Leukocyte disorders have recently gained some interest as biomarkers, following data reporting leukocytosis would occur in 1–10% of patients with nonhematopoietic malignancies and would be associated with a poorer prognosis [9, 15–17]. Leukocytosis is caused by upregulation of expression of hematological growth factors produced by tumors. Those, which act as autocrine stimulating agents, include granulocyte colony–stimulating growth factors, granulocyte macrophage colony-stimulating growth factor, interleukins (1 and 6) and tumor-necrosis factor alpha (TNFα) [16, 18, 19]. Mabuchi et al. examined the prognostic value of leukocytosis in a cohort of cervical cancer patients. They retrospectively found that a white blood cells count ≥ 10,000/μl was associated with more frequent treatment failures and shorter survival. They also reported that tumors from patients with leukocytosis had stronger immunoreactivity for G-CSF than those obtained from patients without leukocytosis. Leukocytosis was a significant prognostic factor for OS in multivariate analysis. This effect on survival was confirmed in a prospective study showing that patients with leukocytosis had more frequent treatment failures, shorter PFS and higher serum G-CSF concentration than those without leukocytosis. When examining leukocyte subtypes, all patients with leukocytosis had elevated neutrophils count, but no increase in basophils or monocytes, indicating that leukocytosis is a result of elevated neutrophils count [9]. More recently, further preclinical investigations were performed to examine the mechanisms responsible for tumor-related leukocytosis in cervical cancer, showing that the increased expression of tumor-derived G-CSF in some cervical cancer was associated with increased frequency of circulating MDSCs in the blood. These cells, which contribute to tumor angiogenesis, metastatic process and immune suppression, could be responsible for the rapidly progressive and radioresistant phenotype of tumor with neutrophilia [11, 12, 18]. Leukocytosis could also reflect a chronic oncogenic inflammation. Actors of this inflammatory process, such as tumor-associated macrophages (TAMs) or MDSCs and their secreted cytokines (IL-6, IL-1β, TNF) are now considered as key in promoting tumor progression [11]. In advanced tumors or in hypoxic regions of tumor stroma, TAMs polarize to type 2 macrophages inhibiting anticancer immunity through the release of factors encouraging recruitment of regulatory T cells as well as production of transforming growth factor β1 (TGF-β) [11]. Furthermore, it has been shown a radiation-induced accumulation of TAMs within 1–2 weeks after treatment, partially as a result of increased levels of the transcription factor HIF-1 (hypoxia inducible factor – 1), potentially resulting in the increased production of proangiogenic factors involved in vascular network recovery and tumor regrowth after irradiation [20].

Although previously described clinical studies provided meaningful information on the prognostic value of tumor-related neutrophilia and the underlying biological mechanisms, they did not allow drawing conclusions on the possibility to integrate this biological information in the treatment process. Patterns of failure were not examined and authors did not examine whether patients experienced more local relapses or more distant metastatic events. Moreover, patients with metastatic disease were included and treatments were not conducted according to modern standards based on chemoradiation plus IGABT for locally advanced cancers, as stage IB2 - IIB carcinomas were treated with upfront radical hysterectomy +/− adjuvant radiotherapy. Finally, no data was provided on technique of brachytherapy and the effect of HR-CTV volume was not examined, while it is a major prognostic factor of patients' outcome in terms of local relapses as well as in terms of distant failures [9, 10, 21]. Consequently, it was not possible, based on previous data from literature, to have any indication on how leukocytosis might be included as part of the decision process in patients who are treated with IGABT.

In the present study, with all patients being prospectively treated with curative intent according to a modern strategy of dosimetric optimization based on MRI findings, we found that neutrophilia was a major prognostic factor for local failures in multivariate analysis, even after taking into account the three main well known prognostic factors for local failure that are tumor size at diagnosis, HR-CTV volume and anemia. The systematic delivery of concurrent chemotherapy and the implementation of dose escalation could account for the lack of significant effect on survival in multivariate analysis, contrary to previous reports examining the prognostic significance of neutrophilia [9]. To our best knowledge, this is the first study reporting the prognostic value of neutrophilia in terms of local relapse. The effect of the D90 HR-CTV was not significant but we have previously shown a significant correlation between the D90 HR-CTV and the risk of local failure in a larger cohort of 225 patients, with a higher statistical power to show this effect [5]. The lack of correlation between dosimetric parameters and probability of local control could be also explained by an advanced optimization process that had been performed in this homogeneous cohort, with a high median D90 HR-CTV dose in most patients. The correlation between the risk of local relapse and presence of neutropenia suggests that neutrophilia could be a valuable and affordable biomarker for improving selection of appropriate candidates to dose escalation, because of a higher risk for local relapse. Another perspective is to conceive pharmacological strategies based on modulation of the tumor immune micro environment to potentiate radiotherapy in these high risk patients, who exhibit radiation-resistant tumors [22, 23]. However, these findings still remain to be confirmed in an independent prospective cohort before applying these concepts to clinics [24]. In particular, the possibility to modulate treatment delivery according to neutrophilia, through dose escalation or dose de-escalation, has not been validated yet.

## MATERIALS AND METHODS

### Patients and tumors

We examined clinical records of consecutive patients treated in our institution between April 2009 and July 2015 for a histologically confirmed locally advanced cervical cancer and receiving concurrent chemoradiation followed by magnetic resonance imaging (MRI)-guided adaptive pulse-dose rate (PDR) brachytherapy within the frame of international prospective observational study examining the effects of MRI-guided brachytherapy in locally advanced cervical cancer: EMBRACE. EMBRACE study aims at assessing MRI-guided brachytherapy for cervical cancer and examining dosimetric parameters of importance for local control and morbidity. All patients had 18-Fluorodeoxyglucose Positron Emission Tomography/Computed tomography (PET/CT) as part of their primary staging. Those without evidence of para-aortic lymph node metastases underwent a primary laparoscopic para-aortic lymphadenectomy.

### Treatment characteristics

Patients received pelvic external beam radiotherapy (EBRT), 45 Gy in 25 fractions of 1.8 Gy through a 3D conformal technique with high megavoltage photons of a linear accelerator. Patients with PET positive para-aortic lymph nodes or histological evidence of para-aortic metastases received extended field radiotherapy. Patients with visceral metastases were excluded. Concurrent chemotherapy was delivered, cisplatin 40 mg/m^2^ weekly. Carboplatin AUC (Area Under Curve) 2 was preferred in case of contraindication to cisplatin.

The PDR brachytherapy boost was based on a MRI computerized assisted treatment planning The vaginal mold technique was used, as previously described [25]. Definitions of volumes followed the guidelines of GEC-ESTRO (*Groupe Européen de Curiethérapie*, European Society for Radiotherapy & Oncology) [26, 27]. Brachytherapy was delivered through one single application per patient. When endocavitary implantation was not sufficient for an adequate coverage of HR-CTV, an additional implantation could be performed with interstitial implantation. Treatment planning objective was to deliver at least 60 Gy to 90% of the intermediate risk clinical target volume (IR-CTV), taking into account the dose delivered by EBRT after converting doses into biological effective doses normalized to a radiobiologically weighted dose equivalent of 2 Gy/fraction (α/β = 10 Gy), and 85 Gy to the D90 of the HR-CTV. Further details on the brachytherapy procedure have been previously reported [9, 12, 25, 26].

Patients with PET positive pelvic or para-aortic lymph nodes received a sequential fractionated boost to deliver 60 Gy to macroscopic lymph nodes, taking into account the contribution of IGABT. The overall treatment time was aimed at not exceeding 55 days.

### Definition of leukocyte disorders

Patients underwent systematic complete blood cell counts weekly during chemoradiation. As this is retrospective analysis, assays were performed blinded to the study end point. Pretreatment blood samples taken before any chemotherapy were employed in the current analysis. Leukocytosis and neutrophilia were defined as a leukocyte count or a neutrophils count exceeding 10,000 and 7,500/μl, respectively. Anemia was defined with threshold of 12 g/dL. These cutpoints were defined from usual thresholds used routinely for defining these blood cell counts disorders. Were excluded from analysis patients who received neoadjuvant chemotherapy, corticosteroids, presenting chronic inflammation, patients treated for an immune disease, or presenting acute or chronic infection (including Human Immunodeficiency Virus).

### Follow-up and statistical analysis

Follow-up was scheduled at 6 weeks, then every three months during two years. A systematic MRI of the pelvis was performed 6–8 weeks after IGABT, then every six months. Hysterectomy was performed only in case of isolated local failure and after a complete restaging through PET/CT. All relapses were considered (not only first relapse) and classified as local failure occurring in the true pelvis, in-field relapses occurring within pelvic and/or para-aortic EBRT fields (if para-aortic area was irradiated), or out-of field relapse (distant metastatic failure). Factors associated with tumor relapse were examined. Survival times were calculated from the time of histological diagnosis and survival rates were estimated using the Kaplan Meier method. Univariate analyses were carried out using log rank tests. Interaction between factors was tested. Multivariate analyses were performed for variables with *p* value < 0.2 in univariate analysis, according to the Cox method. Statistical analyses were performed using SPSS statistics 20.0^®^ (Statistical Package for Social Science) for windows (an IBM company software, Chicago, Illinois, USA).

## CONCLUSIONS

In this cohort of patients treated homogeneously according to modern standards, we found that neutrophilia was a significant prognostic factor for local relapse in locally advanced cervical cancer treated with MRI-based IGABT. This biomarker could be used in further clinical trials to better identify patients with higher risk of local relapse and requiring dose escalation and/or concurrent administration of biological modifiers.
